# Evidence of a founder *BRCA1* mutation in Scotland

**DOI:** 10.1054/bjoc.1999.0984

**Published:** 2000-01-18

**Authors:** A Liede, B Cohen, D M Black, R H Davidson, A Renwick, E Hoodfar, O I Olopade, M Micek, V Anderson, R De Mey, A Fordyce, E Warner, J L Dann, M-C King, B Weber, S A Narod, C M Steel

**Affiliations:** University of Toronto, Breast Cancer Research, 790 Bay Street, Suite 750A, Ontario, Toronto, M5G 1N8, Canada; University of St. Andrews, School of Biological and Medical Sciences, Bute Medical Building, St Andrews, Fife, KY16 9TS, UK; University of Glasgow, Cancer Molecular Genetics Group, Beatson Institute for Cancer Research, Bearsden, Glasgow, G61 1BD, UK; University of Chicago Medical Center, Cancer Risk Clinic, 5841 South Maryland, Chicago, Illinois, 60637-1470, USA; Section of Genetics, Department of Laboratory Medicine, University of Victoria, 35 Helmcken Road, Victoria, British Columbia, V8Z 6R5, Canada; Division of Medical Oncology, Toronto-Sunnybrook Regional Cancer Centre, 2075 Bayview Avenue, Toronto, Ontario, M4N 3M5, Canada; Department of Medicine and Genetics, K160 Health Sciences Center, University of Washington, Box 357720, Seattle, Washington, 98195-7720, USA; Departments of Medicine and Genetics, University of Pennsylvania, 3400 Spruce Street, Philadelphia, Pennsylvania, 19104, USA

**Keywords:** BRCA1, BRCA2, breast cancer genetics, founder BRCA1/2 mutation, Scotland, genotype analysis, founder effect, breast-ovarian cancer syndrome

## Abstract

*BRCA1* mutations have been identified in breast and ovarian cancer families from diverse ethnic backgrounds. We studied 17 different families with the *BRCA1* 2800delAA mutation; seven were ascertained in Scotland (Dundee, Edinburgh, Glasgow, St Andrews), five in Canada (Toronto, Victoria) and five in the United States (Chicago, Philadelphia, Seattle). Overall there was a clear preponderance of Scottish ancestry. Genotype analysis performed on key members from 17 families was consistent with a common haplotype, strongly suggesting a single ancestral origin. A possible link was established between two families by tracing their genealogies through the records of the Registrar General for Scotland. This is the first example of a *BRCA1* mutation likely to be derived from a common founder in Scotland. Further studies will be necessary to estimate more accurately the population frequency of the *BRCA1* 2800delAA mutation among unselected cases of breast and ovarian cancer in Scotland and the UK. © 2000 Cancer Research Campaign


British Journal of Cancer (2000) **82**(3), 705–711
© 2000 Cancer Research Campaign
DOI: 10.1054/ bjoc.1999.0984, available online at http://www.idealibrary.com on IDEAL®

### Evidence of a founder *BRCA1* mutation in Scotland

**A Liede[1], B Cohen[2], DM Black[3], RH Davidson[3], A Renwick[3], E Hoodfar[1], OI Olopade[4], M Micek[5], V Anderson[2], R De Mey[2], A Fordyce[2], E Warner[6], JL Dann[7], M-C King[7], B Weber[8], SA Narod[1] and CM Steel[2]**

[1]University of Toronto, Breast Cancer Research, 790 Bay Street, Suite 750A, Toronto, Ontario M5G 1N8, Canada; [2]University of St. Andrews, School of Biological and Medical Sciences, Bute Medical Building, St Andrews, Fife KY16 9TS, UK; [3]University of Glasgow, Cancer Molecular Genetics Group, Beatson Institute for Cancer Research, Bearsden, Glasgow G61 1BD, UK; [4]University of Chicago Medical Center, Cancer Risk Clinic, 5841 South Maryland, Chicago, Illinois 60637-1470, USA; [5]University of Victoria, Section of Genetics, Department of Laboratory Medicine, 35 Helmcken Road, Victoria, British Columbia V8Z 6R5, Canada; [6]Toronto-Sunnybrook Regional Cancer Centre, Division of Medical Oncology, 2075 Bayview Avenue, Toronto, Ontario M4N 3M5, Canada; [7]University of Washington, Department of Medicine and Genetics, K160 Health Sciences Center, Box 357720, Seattle, Washington 98195-7720, USA; [8]University of Pennsylvania, Departments of Medicine and Genetics, 3400 Spruce Street, Philadelphia, Pennsylvania 19104, USA

**Summary** *BRCA1* mutations have been identified in breast and ovarian cancer families from diverse ethnic backgrounds. We studied 17 different families with the *BRCA1* 2800delAA mutation; seven were ascertained in Scotland (Dundee, Edinburgh, Glasgow, St Andrews), five in Canada (Toronto, Victoria) and five in the United States (Chicago, Philadelphia, Seattle). Overall there was a clear preponderance of Scottish ancestry. Genotype analysis performed on key members from 17 families was consistent with a common haplotype, strongly suggesting a single ancestral origin. A possible link was established between two families by tracing their genealogies through the records of the Registrar General for Scotland. This is the first example of a *BRCA1* mutation likely to be derived from a common founder in Scotland. Further studies will be necessary to estimate more accurately the population frequency of the *BRCA1* 2800delAA mutation among unselected cases of breast and ovarian cancer in Scotland and the UK. © 2000 Cancer Research Campaign

**Keywords:** BRCA1; BRCA2; breast cancer genetics; founder BRCA1/2 mutation; Scotland; genotype analysis; founder effect; breast-ovarian cancer syndrome

Up to 7% of breast cancer cases in North America are estimated to be due to mutations in predisposing breast cancer genes (Claus et al, 1996; Newman et al, 1998). It is estimated that from 1 in 850 to 1 in 500 individuals carry a mutation in either of the highly penetrant breast and ovarian cancer susceptibility genes *BRCA1* or *BRCA2* (Easton et al, 1993; Ford et al, 1995). Women who carry a mutation in the *BRCA1* gene have a 56–87% risk of developing breast cancer and a 16–60% risk of developing ovarian cancer by age 70 (Ford et al, 1994; Easton et al, 1995; Struewing et al, 1997). Over 300 different mutations in the *BRCA1* gene have been reported to the Breast Cancer Information Core (BIC) database (BIC, 1998), but the number of different mutations in a defined ethnic group may be restricted. Although more are anticipated, specific populations for which mutations in *BRCA1* and *BRCA2* have been described include Austria, Netherlands and Belgium, Iceland, Sweden, Hungary, Russia and other Eastern European nations, as well as the Ashkenazi Jewish and French Canadian populations (Johannesdottir et al, 1996; Neuhausen et al, 1996; Tonin et al, 1996, 1998; Gayther et al, 1997; Peelen et al, 1997; Petrij-Bosch et al, 1997; Ramus et al, 1997; Szabo and King, 1997). The most common *BRCA1* mutation among Europeans, the 5382insC mutation, was found in about 79% of breast–ovarian cancer families in Russia (Gayther et al, 1997). The *BRCA1* 5382insC mutation was previously found in a West Lothian (South East) kindred and in an isolated case of breast cancer in Scotland (Mullen et al, 1997). The mutant haplotype was found to be identical to that reported in all other apparently unrelated breast–ovarian cancer families across Europe, with the exception of marker D17S1327 for the West Lothian family. This may represent a different origin for the West Lothian family than the Baltic region than other 5382insC families (Mullen et al, 1997).

Frequent mutations in an ethnically restricted population are due to a founder effect. Founder effects may be most prominent in isolated populations that undergo rapid expansion from a limited number of ancestors. The most frequently reported mutation in the BIC database is the *BRCA1* 185delAG (BIC, 1999). This mutation is estimated to be present in 1% of the Ashkenazi Jewish population and has been reported in North America, Israel, Iran, Iraq and Europe (Struewing et al, 1995; Roa et al, 1996; Szabo and King, 1997). The reconstruction of haplotypes bearing common mutations, such as the 185delAG, provided evidence for strong founder effects (Simard et al, 1994; Neuhausen et al, 1996). In the UK, the 185delAG mutation was found in three Yorkshire families (Neuhausen et al, 1996; Xu et al, 1997). The Yorkshire families were found to share a haplotype that differed from the Ashkenazi Jewish haplotype (Neuhausen et al, 1996; Xu et al, 1997). This was evidence of a separate origin or founder for the *BRCA1* 185delAG mutation in the Ashkenazi Jewish population and the Yorkshire families.

The *BRCA1* 2800delAA mutation was first reported in 1994 in a breast-ovarian cancer family of Scottish descent in the US (Friedman et al, 1994). In 1995, Boyd et al described a Glasgow breast cancer patient as homozygous for the 2800delAA mutation. More recently, Peto et al (1999) studied the prevalence of *BRCA1*

*Received 26 February 1999*
*Revised 3 June 1999*
*Accepted 28 June 1999*

*Correspondence to:* A Liede

**Table 1** BIC database summary sheet for >8 entries of suspected British origin (November 1999)

| *BRCA1* mutation | Exon | Entries | Centres | Ethnicity |
|---|---|---|---|---|
| G546T (E143X) | 7 | 10 | 1 | Irish, Western Europe |
| 1294del40 | 11a | 15 | 6 | British, Irish |
| 2080delA | 11b | 12 | 6 | Dutch, British |
| 2800delAA | 11c | 11 | 6 | British, Scottish |
| T3053G (Y978X) | 11c | 9 | 4 | Irish, Iraqi |
| 3875del4 | 11d | 26 | 11 | Dutch, British, Czech |
| 4184del4 | 11d | 30 | 12 | British, French, Indian, Pakistani |
| C4446T (R1443X) | 13 | 38 | 13 | British, French, Fr. Can., Belgian, Dutch |

and *BRCA2* mutations in an unselected series of early-onset breast cancer patients (under age 46), regardless of family history. Peto et al (1999) detected the *BRCA1* 2800delAA mutation in a patient from Glasgow. To explore the possibility that the 2800delAA represents a population-specific *BRCA1* mutation, we collected information and DNA specimens on 17 apparently unrelated BRCA1 2800delAA families across North America and Scotland. Genotype analysis was performed using five polymorphic microsatellite markers on chromosome 17q to determine whether this mutation represents a founder effect.

### SUBJECTS AND METHODS

### Identification of families with 2800delAA

The Breast Cancer Information Core Database (1997, 1998) was searched for all entries with the *BRCA1* 2800delAA mutation. At the time, nine entries were found from four different centres for the 2800delAA (Table 1). Communication with genetics clinics in North America identified four additional families. The majority of families were tested through clinical service following referral for genetic counselling and risk assessment. These families typically had evidence of genetic predisposition to breast cancer (e.g. two first- or second-degree relatives diagnosed with breast cancer below age 50 or ovarian cancer). One Toronto family (UT10872) was identified by a population-based study of unselected ovarian cancer cases in the province of Ontario. One Scottish family (USt97) was among fifty unselected breast cancer cases diagnosed below age 50 in South East Scotland. Three families had been reported previously; two from Seattle (UW1, UW109) (Friedman et al, 1994) and one from Glasgow (UG475) (Boyd et al, 1995).* Where possible, cancer diagnoses were confirmed by retrieval of pathology or medical records. Detailed family history information was collected for each kindred.

### Controls

Control DNA was obtained from healthy Scottish blood donors via the South Eastern Scotland Blood Transfusion Service. In addition, 50 unrelated individuals (e.g. spouses from within affected families) were tested for the 2800delAA.

*Like USt97, she was ascertained through a survey of early onset breast cancers and, like US97, she has no known family history of breast or related cancers. Hence the penetrance of this mutation may be overestimated by selecting cases on the basis of family history. We are grateful to the authors of that report for their cooperation in providing additional information.

### Mutation detection

Like the majority of *BRCA1* mutations reported thus far, the 2800delAA leads to truncation of the mutant protein and predicted loss of function. A variety of methods can be applied to initially detect the presence of a *BRCA1* mutation (Friedman et al, 1995). The deletion of two adenine nucleotides in the *BRCA1* sequence of exon 11 (2800delAA) results in an in-frame stop codon at nucleotide 2820. The chain-terminating mutation in exon 11 may be screened by protein-truncation test (PTT) or by single-strand conformational analysis (SSCA) or by a restriction enzyme assay as the mutation creates a new Tth recognition site. Mutant bands were confirmed with direct sequencing of the exon 11 region.

### Genotyping markers in the *BRCA1* region

Human genomic DNA was isolated from 20–30 ml EDTA or ACD blood samples. Five polymorphic microsatellite markers located on chromosome 17q either within *BRCA1* or within 250 kb of it were used to construct genotypes (Friedman et al, 1994). These markers were used in non-radioactive polymerase chain reaction (PCR) using 20 cycles, run on sequencing-type gel electrophoresis, then transferred to nylon membranes blotted with 32P labelled oligo (CA) or with a 32P labelled primer if the locus was not a dinucleotide repeat. From centromere to telomere, the order reported in the Genome Database (GDB) is as follows: D17S1321, D17S855, D17S1322, D17S1323, D17S1327. The total distance covered by these five markers is 405 kb. Primer sequences to amplify these markers can be retrieved on-line from the GDB. A physical map for these markers has been previously resolved (Neuhausen et al, 1996): D17S1321-(255 kb)-D17S855-(5 kb)-D17S1322-(45 kb)-D17S1323-(100 kb)-D17S1327

### Registrar General for Scotland

Complete and informative records of all Scottish births, marriages and deaths, dating from the introduction of compulsory registration in 1855, are held in Register House, Edinburgh, along with many much older, though incomplete, genealogical data sets. These are public records, so that the construction of accurate multi-generation family trees presents fewer problems than is the case in many countries (Collyer and De Mey, 1987; Sinclair, 1990). Using these records we explored the possibility of an ancestral link between the 17 families. Two of these families are thought to be related as they are known to be descended from ancestors with the same unusual surname, born in the same part of Scotland around 1850. Other families were not linked through genealogy records.

 © 2000 Cancer Research Campaign

**Table 2** Characteristics of 17 families with *BRCA1* 2800delAA mutations

| | Family | Centre | Ancestry | Breast cancer | | | | | Ovarian cancer | | Primaries | Other cancers |
|---|---|---|---|---|---|---|---|---|---|---|---|---|
| | | | | Total | ≤50 | >50 | Bilateral Br | Av. age | Total | Av.Age | Br/Ov | |
| 1 | UT271 | Toronto | Scotland | 4 | 3 | 1 | 0 | 44 (27–55) | 0 | | 0 | Prostate (2) |
| 2 | UT9151 | Toronto | Scotland | 3 | 2 | 1 | 0 | 46 (38,45,55) | 0 | | 0 | Rhabdomyosarcoma (1), Testes (1), Skin (1) |
| 3 | UT9213 | Toronto | Britain | 3 | 3 | 0 | 0 | 39 (28,42,46) | 0 | | 0 | Prostate (2), Bladder (1), Cervix (2) |
| 4 | UT10872 | Toronto | Scotland | 5 | 5 | 0 | 0 | 48 (45–50) | 1 | 53 | 1 | None recorded |
| 5 | UV5763 | Victoria | Scotland | 4 | 2 | 2 | 0 | 52 (40–75) | 0 | | 0 | Prostate (2) |
| 6 | UW1 | Seattle | Britain | 16 | 14 | 2 | 1 | 38 (25–74) | 1 | 61 | 1 | Prostate (2), Colon (2), Fallopian Tube (1) |
| 7 | UW109 | Seattle | Scotland | 4 | 3 | 1 | 1 | 44 (32–51) | 2 | 49 (47–52) | 2 | Prostate (1), Stomach (1), Lung (1), Lymphoma (1) |
| 8 | UC9214 | Chicago | Scotland | 6 | 5 | 1 | 0 | 41 (29–60) | 2 | 56 (48,65) | 0 | Colon (2), Melanoma (1), Leukaemia (1) |
| 9 | UP335 | Philadelphia | Britain | 3 | 3 | 0 | 0 | 39 (33,40,43) | 2 | 44 (39,49) | 1 | Lung (1) |
| 10 | UP472 | Philadelphia | Britain | 10 | 3 | 1 | 1 | 42 (37–52) | 1 | | 0 | Prostate (1), Spinal (1), Stomach (1), Skin (1) |
| 11 | UE2 | Edinburgh | Scotland | 8 | 3 | 5 | 3 | 58 (37–69) | 3 | 45 (42,45,47) | 3 | Prost (1), Col (4), Lung (2), Oesoph (1), Blad (1), Astrocyt (1) |
| 12 | UD90325 | Dundee | Scotland | 3 | 3 | 0 | 1 | 34 (25,36,41) | 2 | 51 (42,60) | 0 | Colon (1), Erythroleukaemia (1) |
| 13 | USt97 | St.Andrews | Scotland | 1 | 1 | 0 | 0 | 43 | 0 | | 0 | Stomach (2), Epiglottis (1), Carcinoid (1), Skin (1), Lung (1) |
| 14 | USt163 | St.Andrews | Scotland | 4 | 4 | 0 | 1 | 35 (27–47) | 4 | 53 (46–62) | 1 | Colon (2), Penis (1) |
| 15 | UG11493 | Glasgow | Scotland | 7 | 5 | 2 | 1 | 45 (34–77) | 0 | | 0 | None recorded |
| 16 | UG13465 | Glasgow | Scotland | 3 | 2 | 1 | 1 | 45 (40,44,51) | 2 | 45 (40,51) | 1 | Prostate (1), Uterus (1), Skin (1) |
| 17 | UG475 | Glasgow | Scotland | 4 | 2 | 2 | 1 | 43 (32–58) | 3 | 43 (40,42,45) | 1 | Lung (2), Mouth (1) |
| | Mean | | | 5.18 | 3.71 | 1.12 | 0.65 | 43 | 1.35 | 57 | 0.65 | |

### RESULTS

Each of the 17 families contained at least two cases of breast cancer diagnosed at age 50 or below, or ovarian cancer at any age, with the exception of family USt97 with only one case of premenopausal breast cancer confirmed (Table 2). The average age of onset of breast cancer was 43 (range 25–77) and the average age of ovarian cancer was 57 (range 39–65). On average, there were five women affected with breast cancer observed per family. Nine of the 17 families had bilateral breast cancer cases. There were no cases of male breast cancer reported in any family. Ten of the 17 families contained cases of ovarian cancer (range 1–3 cases). Eight of these ten ovarian cancer families contained women diagnosed with primary cancers of the breast and ovary. Family UW1 included one case of primary cancer of the fallopian tube at age 48. Of the 17 families, eight contained prostate cancer, including four families with two cases of prostate cancer. In general, mutation status was not available for members of a family affected with cancers other than breast and ovarian cancer, with the exception of three cases (fallopian tube, colon and prostate cancers in UW1). The characteristics of the 17 families with the 2800delAA mutation are summarized in Table 2. The genotype observed for the 17 families was in concordance with the previous reports by Friedman et al (1994, 1995) for two Seattle families (UW1, UW109). The genotype for the polymorphic microsatellite markers D17S1321-D17S855-D17S1322-D17S1323-D17S1327 was 14–5–5–6–12 for the DNA samples tested belonging to the 17 *BRCA1* 2800delAA families (Figure 1). This genotype was observed for all DNA samples of mutation carriers tested belonging to the 17 families. It was possible to establish phase unambiguously for six families (UW1, UG475, UC9214, UT9213, UT9151, UE2) where multiple family members had participated in genetic studies. Although phase was not established for the remaining 11 families the genotype observed was consistent. Six North American families reported Scottish ancestry. In four of the North American families, the index case reported ancestry from the British Isles.

Seven families from Scotland were among 150 multi-case kindreds tested through the clinical genetics services. Of 50 unselected breast cancer cases presenting under the age of 50 in the South East region, one was found to have the 2800delAA mutation in *BRCA1* (USt97). Of 50 unrelated individuals (e.g. spouses) tested in our series, none had the *BRCA1* 2800delAA mutation. DNA samples collected on population-based controls in Scotland were tested specifically for the *BRCA1* 2800delAA. Molecular analysis did not detect the 2800delAA mutation in any of the 100 non-cancer control samples tested.

Figure 1 depicts a representative *BRCA1* 2800delAA families UV5763, UW109, UC9214, UT9151, UT10872 and UT271. Figure 2 depicts family UG475 previously reported by Boyd et al (1995) with a homozygous *BRCA1* 2800delAA mutant (indicated as proband in pedigree). This family was also found to have a *BRCA2* 8297delC mutation, detected in the maternal cousin of the proband. For Table 2, information regarding the maternal cousin with the *BRCA2* 8297delC mutation was excluded.

### DISCUSSION

The presence of specific *BRCA1* or *BRCA2* mutations is dependent in part on the population from which the breast and ovarian cancer families are ascertained, emphasizing the importance of founder

© 2000 Cancer Research Campaign

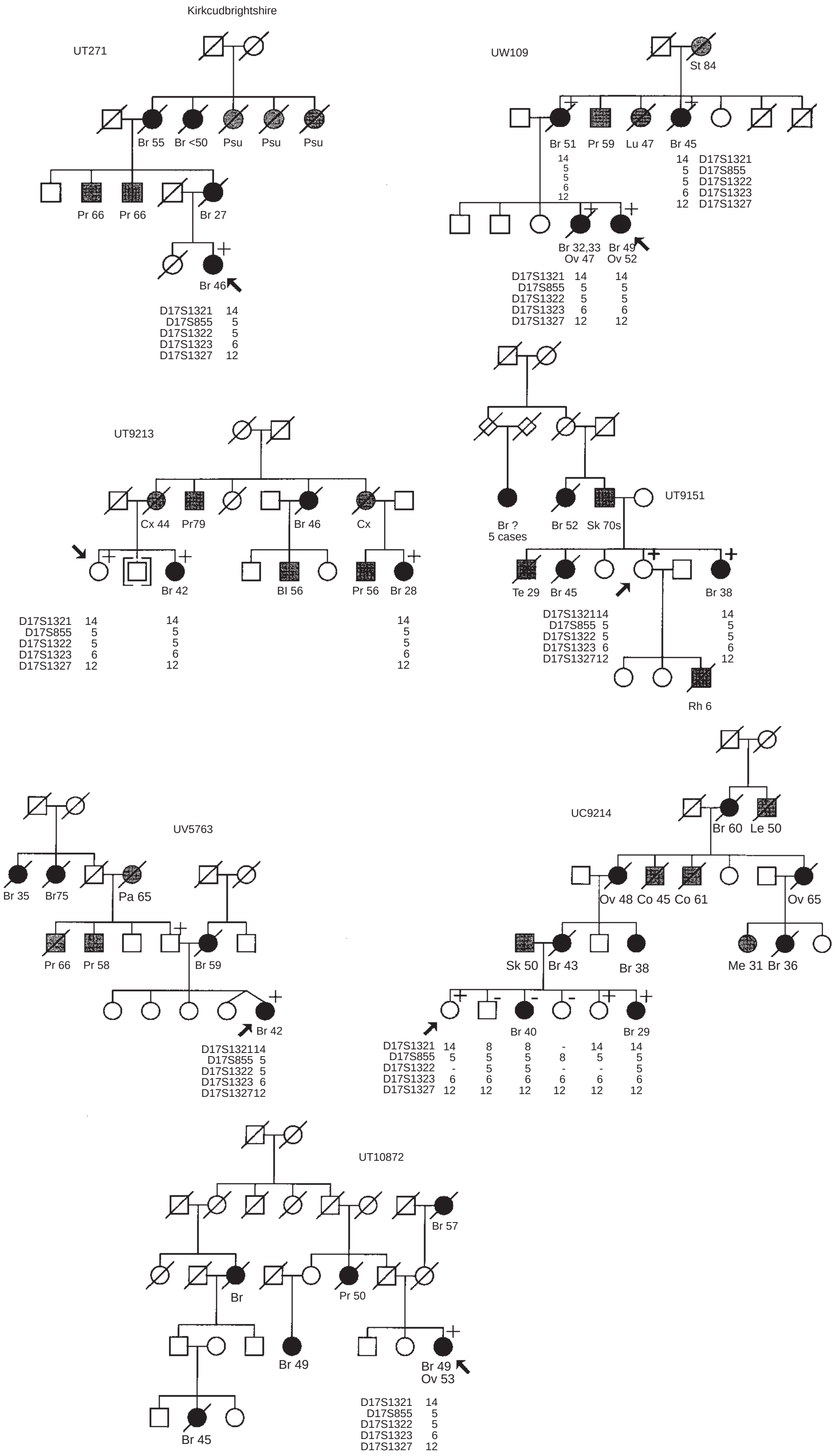

**Figure 1** Pedigrees of families UV5763, UW109, UC9214, UT9151, UT9213, UT10872 and UT271. Black circles indicate women affected with breast or ovarian cancer. Squares indicate men. Grey circles and squares indicate individuals affected with cancers other than breast or ovarian cancer. Diagonal slash indicates deceased. Ov = ovarian cancer, Br = breast cancer, Pr = prostate cancer, Pa = pancreatic cancer, Bl = bladder cancer, Cx = cervical cancer, St = stomach cancer, Lu = lung cancer, Sk = skin cancer, Te = testicular cancer, Rh = rhabdomyosarcoma, Psu = primary site of cancer unknown. The numbers following the abbreviations indicate ages of diagnosis. The plus sign indicates the presence of the *BRCA1* 2800delAA mutation. The associated haplotype for polymorphic microsatellite markers D17S1321–D17S855–D17S1322–D17S1323–D17S1327 of 14–5–5–6–12 is indicated below the symbols of the individuals genotyped

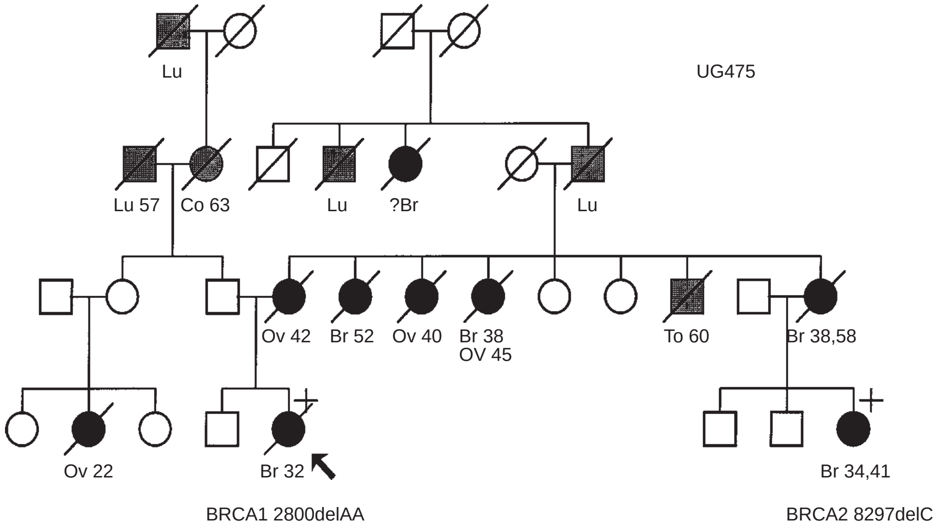

**Figure 2** Pedigree of family UG475. Black circles indicate women affected with breast or ovarian cancer. Squares indicate men. Grey circles and squares indicate individuals affected with cancers other than breast or ovarian cancer. Diagonal slash indicates deceased. Ov = ovarian cancer, Br = breast cancer, Co = colorectal cancer, Lu = lung cancer, To = tongue cancer. The numbers following the abbreviations indicate ages of diagnosis. The plus sign indicates the presence of a *BRCA1* or *BRCA2* mutation (indicated below symbol)

mutations in population-specific studies (Neuhausen et al, 1996; Szabo and King, 1997). In particular, haplotype and genotype analysis provides evidence that a specific *BRCA1* or *BRCA2* mutation can be associated with the geographic or ethnic origin of the study population. This knowledge is relevant not only for the country of origin of the common founder, but also for immigrant populations. Many European *BRCA1* and *BRCA2* mutations have been observed in the USA or Canada, reflecting migration patterns to North America.

The most common *BRCA1* mutations found in British breast-ovarian cancer families to date have been the 1294del40, 4184del4, C4446T, 3875del4 and 2800delAA mutations (Table 1). There were between 11 and 38 entries for each of these *BRCA1* mutations in the BIC Database (BIC, 1999). The C4446T mutation was reported frequently and families were of British, Belgian, French and French-Canadian descent (Neuhausen et al, 1996; Tonin et al, 1998). The 4184del4 mutation is reported in numerous families in North America and Europe (BIC, 1998). Previous genotype analysis for *BRCA1* 4184del4 mutation families found diversity in the haplotypes which was not surprising considering the families were of British, French, Indian and Pakistani descent (Friedman et al, 1995; Neuhausen et al, 1996; Szabo and King, 1997). In Wales, four *BRCA1* (4184del4, 2011insT, 2594delC, 1997del4) and two *BRCA2* (4075delGT, 6287del4) mutations were detected in eight breast cancer families, including two families with the *BRCA1* 4184del4 mutation and two families with the 2594delC mutation (Lancaster et al, 1998). The 2080del4 mutation (Table 1) has been reported in several Dutch families and, more recently, in two breast cancer patients of the unselected series in the UK by Peto et al (1999). Other mutations include the *BRCA1* G2508T and the *BRCA2* 3295insA, both found in a breast cancer patient of Scottish descent (Liede et al, 1998). Only the 1294del40 and the 2800delAA mutations have been reported exclusively in British and North American families. There were 15 entries for the *BRCA1* 1294del40 mutation found in six centres in Britain and North America (BIC, 1999). Neuhausen et al (1996) demonstrated a conserved haplotype for six 1294del40 mutation families which implied a common ancestral origin for the 1294del40 families of British-Irish descent. Friedman et al (1994) first reported the *BRCA1* 2800delAA mutation. The 2800delAA was also previously found in Scottish families, including an individual apparently homozygous for the 2800delAA mutation (Boyd et al, 1995). In this study of 17 distinct *BRCA1* 2800delAA families, we provide evidence for a founder *BRCA1* mutation specific to the Scottish population.

The Scottish population is frequently described as being of 'Celtic' origin though the definition of Celtic is somewhat imprecise. The Celts are believed to have spread from the mainland of Europe via Northern France and Spain to Cornwall, Wales, Ireland and Scotland, beginning over 2000 years ago. There were indigenous Scots who were displaced by, or who merged with, the incoming Celts and in the intervening centuries Viking raiders and other Scandinavian seafarers and fisherfolk have also added to the population mix. In the study of breast–ovarian cancer families in Wales, the *BRCA1* 2800delAA was not detected among 17 families (Lancaster et al, 1998). However, culturally and linguistically the Irish and Scottish Celts appear to have more in common with each other than with present day Bretons, Cornish or Welsh. Previous studies on the distribution of genetic polymorphisms, such as HLA haplotypes, tend to place the Irish/Scottish 'Celts' somewhere between the modern day English and the Scandinavian populations (Bodmer et al, 1987).

The present day Scottish population has expanded over the past 150 years from a somewhat depleted gene pool. The tendency of both Scots and Irish to establish themselves in all parts of the world is well recognized. There has been relatively little inward migration in the past 200 years but a very substantial emigration, principally to North America and Australia, during the mid nineteenth century, coinciding with the Highland 'clearances' when large sections of the population were displaced from land they occupied as tenant farmers. Several of the families ascertained in Scotland for this study included relatives in North America and Australia who have obtained counselling and screening as a result of contacts maintained with their roots. Genotype and limited

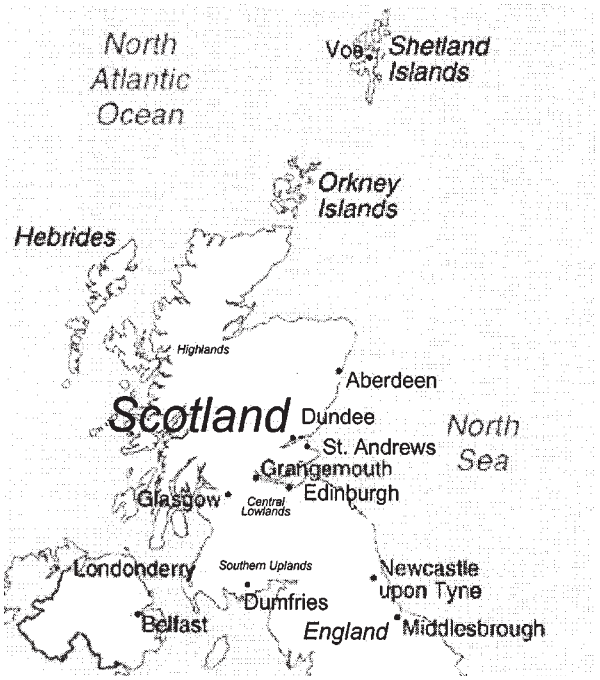

**Figure 3** Map of Scotland. The *BRCA1* 2800delAA families likely originated from the Central Lowlands and Southern Uplands of Scotland. The *BRCA1* 2800delAA families were identified through regional genetics clinics in the West Central region of Scotland (Glasgow, Edinburgh, Dundee and St Andrews). One Toronto family (UT271) originated from the county of Kirkudbrightshire (near Dumfries). To date, the 2800delAA mutation has not been identified in families from the Aberdeen catchment area of the North East (Haites, personal communication). Map was modified from Virtual Tourist web-site: http://www.vtourist.com/Europe/United_Kingdom/ciafacts haplotype analysis for key individuals belonging to families with the *BRCA1* 2800delAA mutation showed that the 17 mutation-positive families share the same *BRCA1*-linked haplotype. Specifically, the ten *BRCA1* 2800delAA families from North America were found to share the Scottish *BRCA1* haplotype. These included five families with reported Scottish ancestry and four families where the index case reported 'British' ancestry when less certain of the geographic origin of their family beyond the British isles or 'Anglo-Saxon' ethnicity (Table 2). The shared haplotype found between these families and the Scottish 2800delAA families supports a common ancestral origin.

Most of the *BRCA1* 2800delAA families appear to originate from West Central Scotland (Figure 3). While 2800delAA families have been identified through regional genetics clinics in Glasgow, Edinburgh and Dundee/St Andrews, to date, the 2800delAA mutation has not been identified in families from the Aberdeen catchment area of the North East (Haites, personal communication). The reverse tendency was noted for Huntington's disease, where a concentration of affected families was traced to the North East and attributed to a common founder, possibly of Scandinavian fisher stock, who settled in Scotland some 300 years ago (Lyon, 1962).

The woman reported by Boyd et al (1995) as a homozygous mutant for the 2800delAA mutation is the proband of family UG475 (Figure 2). We did not re-examine homozygous status of the proband in this study. Interestingly, the proband's maternal cousin diagnosed with bilateral breast cancer does not carry the *BRCA1* 2800delAA mutation, instead she was found to have a *BRCA2* 8297delC mutation. Consequently, it remains unclear which cancer cases in family UG475 are associated with a *BRCA1* or *BRCA2* mutation or both. The *BRCA2* 8297delC is a novel mutation detected in another Glasgow family UG12343 which contains four cases of female breast cancer (ages 37, 47, 64, 64) and one case of prostate cancer.

In Glasgow, Dundee/St Andrews and Edinburgh, a series of breast–ovarian cancer families with evidence for a genetic predisposition (e.g. two first- or second-degree relatives diagnosed with breast cancer below age 50 or ovarian cancer) was tested and six *BRCA1* 2800delAA mutation families were identified. Approximately 4% of at-risk Scottish families were found to have the *BRCA1* 2800delAA mutation. The absence of the 2800delAA mutation in a series of 100 non-cancer controls is consistent with the pathogenic nature of the *BRCA1* mutation.

The overall incidence of breast cancer in Scotland is relatively high; the age-standardized rate (world) for Scotland is 72.70 per 100 000, whereas the rate for England and Wales is 68.84 per 100 000 (IARC, 1997). Information on the prevalence of *BRCA1* and *BRCA2* in breast and ovarian cancer incidence specific to Scotland is not available. Larger population-based studies are necessary to more accurately estimate the contribution of the *BRCA1* and *BRCA2* in the general breast and ovarian cancer incidence in Scotland. Although further studies are necessary, we estimate the frequency of the *BRCA1* 2800delAA mutation among Scottish breast cancer patients diagnosed below age 50 could reach 2%. The overall frequency of this mutation in the general population in Scotland is expected to lie below 1%.

### ACKNOWLEDGEMENTS

We acknowledge the contribution of patients to this study by Drs Elaine Anderson and Marta Reis. We thank Elaine Jack, Karen Robb, Elaine Kwan, Danny Vesprini, Gord Glendon, Joanne Honneyford, Shelly Cummings, Maureen Seminsky and Drs. David Goudie, Doug Horsman and Jackie Yule for technical support. We thank Professor Neva Haites for personal communication and update of the Aberdeen regional genetics service. Dr Mary-Claire King's laboratory is funded by National Institutes of Health grant R01 CA 27632. Alexander Liede is an MRC (Canada) doctoral scholar.

### REFERENCES

BIC (1998) Breast Cancer Information Core Database: *http://www.nhgri.nih.gov/Intramural_research/Lab_transfer/Bic/*

Bodmer JG, Kennedy LJ, Lindsay J and Wasik AM (1987) Applications of serology and the ethnic distribution of three locus HLA haplotypes. *Br Med Bull* **43**: 94–121

Boyd M, Harris F, McFarlane R, Davidson HR and Black DM (1995) A human *BRCA1* gene knockout. *Nature* **375**: 541–542

Claus EB, Schildkraut JM, Thompson WD and Risch NJ (1996) The genetic attributable risk of breast and ovarian cancer. *Cancer* **77**: 2318–2324

Collyer S and De Mey R (1987) Public records and recognition of genetic disease in Scotland. *Clin Genet* **31**: 125–131

Easton DF, Ford D and Peto J (1993) Inherited susceptibility to breast cancer. *Cancer Surv* **18**: 1–17

Easton DF, Ford D and Bishop DT (1995) Breast and ovarian cancer incidence in *BRCA1*-mutation carriers. Breast Cancer Linkage Consortium. *Am J Hum Genet* **56**: 265–271

Ford D, Easton DF, Bishop DT, Narod SA and Goldgar DE (1994) Risks of cancer in *BRCA1* mutation carriers. *Lancet* **343**: 692–695

Ford D, Easton DF and Peto J (1995) Estimates of the gene frequency of *BRCA1* and its contribution to breast and ovarian cancer incidence. *Am J Hum Genet* **57**: 1457–1462

© 2000 Cancer Research Campaign

Friedman LS, Ostermeyer EA, Szabo CI, Dowd P, Lynch ED, Rowell SE and King M-C (1994) Confirmation of *BRCA1* by analysis of germline mutations linked to breast and ovarian cancer in ten families. *Nat Genet* **8**: 399–404

Friedman LS, Szabo CI, Ostermeyer EA, Dowd P, Butler L, Park T, Lee MK, Goode EL, Rowell SE and King M-C (1995) Novel inherited mutations and variable expressivity of *BRCA1* alleles, including the founder mutation 185delAG in Ashkenazi Jewish families. *Am J Hum Genet* **57**: 1284–1297

Gayther SA, Harrington P, Russell P, Kharkevich G, Garkavtseva RF and Ponder BAJ (1997) Frequently occurring germ-line mutations of the *BRCA1* gene in ovarian cancer families from Russia. *Am J Hum Genet* **60**: 1239–1242

IARC (1997) *Cancer Incidence in Five Continents*, Vol. VII. International Agency for Research on Cancer: Lyon

Johannesdottir G, Gudmundsson J, Bergthorsson JT, Arason A, Agnarsson BA, Eiriksdottir G, Johannsson OT, Borg A, Ingvarsson S, Easton DF, Egilsson V and Barkardottir RB (1996) High prevalence of the 999del5 mutation in Icelandic breast and ovarian cancer patients. *Cancer Res* **56**: 3663–3665

Lancaster JM, Carney ME, Gray J, Myring J, Gumbs C, Sampson J, Wheeler D, France E, Wiseman R, Harper P and Futreal PA (1998) BRCA1 and BRCA2 in breast cancer families from Wales: moderate mutation frequency and two recurrent mutations in BRCA1. *Br J Cancer* **78**: 1417–1420

Liede A, Rehal P, Vesprini D, Jack E, Abrahamson J and Narod SA (1998) A breast cancer patient of Scottish descent with germ-line mutations in *BRCA1* and *BRCA2*. *Am J Hum Genet* **60**: 1543–1544

Liu CY, Flesken-Nikitin A, Li S, Zeng Y and Lee WH (1996) Inactivation of the mouse *BRCA1* gene leads to failure in the morphogenesis of the egg cylinder in early postimplantation development. *Genes Dev* **10**: 1835–1843

Lyon RLL (1962) Huntington's chorea in the Moray Firth area. *Br Med J* **i**: 1301–1306

Mullen P, Miller WR, Mackay J, Fitzpatrick DR, Langdon SP and Warner JP (1997) *BRCA1* 5382insC in sporadic and familial breast and ovarian carcinoma in Scotland. *Br J Cancer* **75**: 1377–1380

Neuhausen SL, Mazoyer S, Friedman L, Stratton M, Offit K, Caligo A, Tomlinson G, Cannon-Albright L, Bishop T, Kelsell D, Solomon E, Weber B, Couch F, Struewing J, Tonin P, Durocher F, Narod S, Skolnick MH, Lenoir G, Serova O, Ponder B, Stoppa-Lyonnet D, Easton D, King M-C and Goldgar DE (1996) Haplotype and phenotype analysis of six recurrent *BRCA1* mutations in 61 families: results of an international study. *Am J Hum Genet* **58**: 271–280

Newman B, Mu H, Butler LM, Millikan RC, Moorman PG and King M-C (1998) Frequency of breast cancer attributable to BRCA1 in a population-based series of American women. *J Am Med Assoc* **279**: 915–921

Peto J, Collins N, Barfoot R, Seal S, Warren W, Rahman N, Easton DF, Evans C, Deacon J and Stratton MR (1999) Prevalence of BRCA1 and BRCA2 gene mutations in patients with early-onset breast cancer. Journal of the National Cancer Institute 91: 943–949

Peelen T, van Vliet M, Petruj-Bosch A, Mieremet C, van den Ouweland AMW, Hoervorst F, Brohet R, Ligtenberg MJL, Teugels E, van der Luijt R, van der Hout AH, Gille JJP, Pals G, Jedema I, Olmer R, van Leeuwen I, Newman B, Plandsoen M, van der Est M, Brink G, Hageman S, Arts PJW, Bakker MM, Willems HW, van der Looij E, Neyns B, Bonduelle M, Jansen R, Oosterwijk JC, Sijmons R, Smeets HJM, van Asperen CJ, Meijers-Heijboer H, Klijn JGM, de Greve J, King M-C, Menko FH, Brunner HG, Halley D, van Ommen G-JB, Vasen HFA, Cornelisse CJ, van't Veer LJ, de Knijff P, Bakker E and Devilee P (1997) A high proportion of novel mutations in *BRCA1* with strong founder effects among Dutch and Belgian hereditary breast and ovarian cancer families. *Am J Hum Genet* **60**: 1041–1049

Petrij-Bosch A, Peelen T, van Vliet M, van Eijk R, Olmer R, Drusedau M, Hogervorst FBL, Hageman S, Arts PJW, Ligtenberg MJL, Meijers-Heijboer H, Klijn JGM, H.F.A. V, Cornelisse CJ, van't Veer LJ, Bakker E, van Ommen G-JB and Devilee P (1997) *BRCA1* genomic deletions are major founder mutations in Dutch breast cancer patients. *Nat Genet* **17**: 341–345

Ramus SJ, Kote-Jarai Z, Friedman LS, van der Looij M, Gayther SA, Csokay B, Ponder BAJ and Olah E (1997) Analysis of *BRCA1* and *BRCA2* mutations in Hungarian families with breast or breast-ovarian cancer. *Am J Hum Genet* **60**: 1242–1246

Roa BB, Boyd AA, Vocik K and Richards CS (1996) Ashkenazi Jewish population frequencies for common mutations in *BRCA1* and *BRCA2*. *Nat Genet* **14**: 185–187

Sinclair S (1990) *Tracing your Scottish Ancestors: a Guide to Ancestry Research in the Scottish Record Office*. HMSO: Edinburgh

Struewing JP, Abeliovich D, Peretz T, Avishai N, Kaback MM, Collins FS and Brody LC (1995) The carrier frequency of the BRCA1 185delAG mutation is approximately 1 percent in Ashkenazi Jewish individuals. *Nat Genet* **11**: 198–200

Struewing JP, Hartge P, Wacholder S, Baker SM, Berlin M, McAdams M, Timmerman MM, Brody LC and Tucker MA (1997) The risk of cancer associated with specific mutations of *BRCA1* and *BRCA2* among Ashkenazi Jews. *New Engl J Med* **336**: 1401–1408

Szabo CI and King M-C (1997) Population genetics of *BRCA1* and *BRCA2*. *Am J Hum Genet* **60**: 1013–1020

Thorlacius S, Sigurdsson S, Bjarnadottir H, Olafsdottir G, Jonasson JG, Tryggvadottir L, Tulinius H and Eyfjord JE (1997) Study of a single *BRCA2* mutation with high carrier frequency in a small population. *Am J Hum Genet* **60**: 1079–1084

Tonin P, Weber B, Offit K, Couch F, Rebbeck TR, Neuhausen S, Godwin AK, Daly M, Wagner-Costalos J, Berman D, Grana G, Fox E, Kane MF, Kolodner RD, Krainer M, Haber DA, Struewing JP, Warner E, Rosen B, Lerman C, Peshkin B, Norton L, Serova O, Foulkes WD, Lynch HT, Lenoir GM, Narod SA and Garber JE (1996) Frequency of recurrent BRCA1 and BRCA2 mutations in Ashkenazi Jewish breast cancer families. *Nat Med* **2**: 1179–1183

Tonin PN, Mes-Masson A-M, Futreal A, Morgan K, Mahon M, Foulkes WD, Cole DEC, Provencher D, Ghadirian P and Narod SA (1998) Founder *BRCA1* and *BRCA2* mutations in French Canadian breast and ovarian cancer families. *Am J Hum Genet* **63**: 1341–1351

Xu CF, Chambers JA, Nicolai H, Brown MA, Hujeirat Y, Mohammed S, Hodgson S, Kelsell DP, Spurr NK, Bishop DT and Solomon E (1997) Mutations and alternative splicing of the *BRCA1* gene in UK breast/ovarian cancer families. *Genes Chromosomes Cancer* **18**: 102–110

### NOTE ADDED IN PROOF

Since the work was completed on the 17 breast and ovarian cancer families with the *BRCA1* 2800delAA germline mutation (this issue), we have learned of the existence of three additional families with this mutation. One is a breast cancer patient from Glasgow ascertained in an unselected series of early-onset breast cancer patients in the UK as reported by Peto et al (1999). The second is a breast cancer patient diagnosed at age 35, of Scottish ancestry, reported to the BIC database by a group in London, Ontario. The third subsequent occurrence of the 2800delAA is in a breast cancer patient ascertained in Edinburgh who originated from South West Scotland and Yorkshire (Table 3). We are currently completing further molecular work on these three additional families. There is evidence to support that the *BRCA1* 2800delAA mutation can be traced to a set of ancestors in Scotland.

**Table 3** Three additional families* with *BRCA1* 2800 delAA)

| *Family* | Centre | Origin or ancestry | Breast cancer total | Breast cancer ≤50 | Breast cancer >50 | Ovarian total | Other cancers |
|---|---|---|---|---|---|---|---|
| 1 Peto et al (1999) | Cambridge & Glasgow | Glasgow, Scotland | 1 | 1 | 0 | 0 | none reported |
| 2 Ainsworth et al (pers comm, BIC 1999) | London, Ontario | Scotland & England | 2 | 2 | 0 | 0 | stomach (1), colon (1) |
| 3 Steel et al (pers comm) | Edinburgh | Scotland & Yorkshire | 5 | 2 | 3 | 1 | colon (1), prostate (1), lung (1) |

*Families found after completion of study by Liede et al; genotype analysis currently underway.

*© 2000 Cancer Research Campaign*

## References

[PDF_00622] Bodmer J. G., Kennedy L. J., Lindsay J., Wasik A. M. (1987). Applications of serology and the ethnic distribution of three locus HLA haplotypes.. Br Med Bull.

[PDF_00625] Boyd M., Harris F., McFarlane R., Davidson H. R., Black D. M. (1995). A human BRCA1 gene knockout.. Nature.

[PDF_00627] Claus E. B., Schildkraut J. M., Thompson W. D., Risch N. J. (1996). The genetic attributable risk of breast and ovarian cancer.. Cancer.

[PDF_00629] Collyer S., De Mey R. (1987). Public records and recognition of genetic disease in Scotland.. Clin Genet.

[PDF_00633] Easton D. F., Ford D., Bishop D. T. (1995). Breast and ovarian cancer incidence in BRCA1-mutation carriers. Breast Cancer Linkage Consortium.. Am J Hum Genet.

[PDF_00636] Ford D., Easton D. F., Bishop D. T., Narod S. A., Goldgar D. E. (1994). Risks of cancer in BRCA1-mutation carriers. Breast Cancer Linkage Consortium.. Lancet.

[PDF_00638] Ford D., Easton D. F., Peto J. (1995). Estimates of the gene frequency of BRCA1 and its contribution to breast and ovarian cancer incidence.. Am J Hum Genet.

[PDF_00652] Friedman L. S., Ostermeyer E. A., Szabo C. I., Dowd P., Lynch E. D., Rowell S. E., King M. C. (1994). Confirmation of BRCA1 by analysis of germline mutations linked to breast and ovarian cancer in ten families.. Nat Genet.

[PDF_00655] Friedman L. S., Szabo C. I., Ostermeyer E. A., Dowd P., Butler L., Park T., Lee M. K., Goode E. L., Rowell S. E., King M. C. (1995). Novel inherited mutations and variable expressivity of BRCA1 alleles, including the founder mutation 185delAG in Ashkenazi Jewish families.. Am J Hum Genet.

[PDF_00659] Gayther S. A., Harrington P., Russell P., Kharkevich G., Garkavtseva R. F., Ponder B. A. (1997). Frequently occurring germ-line mutations of the BRCA1 gene in ovarian cancer families from Russia.. Am J Hum Genet.

[PDF_00664] Johannesdottir G., Gudmundsson J., Bergthorsson J. T., Arason A., Agnarsson B. A., Eiriksdottir G., Johannsson O. T., Borg A., Ingvarsson S., Easton D. F. (1996). High prevalence of the 999del5 mutation in icelandic breast and ovarian cancer patients.. Cancer Res.

[PDF_00678] LYON R. L. (1962). Huntington's chorea in the Moray Firth area.. Br Med J.

[PDF_00668] Lancaster J. M., Carney M. E., Gray J., Myring J., Gumbs C., Sampson J., Wheeler D., France E., Wiseman R., Harper P. (1998). BRCA1 and BRCA2 in breast cancer families from Wales: moderate mutation frequency and two recurrent mutations in BRCA1.. Br J Cancer.

[PDF_00672] Liede A., Rehal P., Vesprini D., Jack E., Abrahamson J., Narod S. A. (1998). A breast cancer patient of Scottish descent with germ-line mutations in BRCA1 and BRCA2.. Am J Hum Genet.

[PDF_00675] Liu C. Y., Flesken-Nikitin A., Li S., Zeng Y., Lee W. H. (1996). Inactivation of the mouse Brca1 gene leads to failure in the morphogenesis of the egg cylinder in early postimplantation development.. Genes Dev.

[PDF_00680] Mullen P., Miller W. R., Mackay J., Fitzpatrick D. R., Langdon S. P., Warner J. P. (1997). BRCA1 5382insC mutation in sporadic and familial breast and ovarian carcinoma in Scotland.. Br J Cancer.

[PDF_00683] Neuhausen S. L., Mazoyer S., Friedman L., Stratton M., Offit K., Caligo A., Tomlinson G., Cannon-Albright L., Bishop T., Kelsell D. (1996). Haplotype and phenotype analysis of six recurrent BRCA1 mutations in 61 families: results of an international study.. Am J Hum Genet.

[PDF_00690] Newman B., Mu H., Butler L. M., Millikan R. C., Moorman P. G., King M. C. (1998). Frequency of breast cancer attributable to BRCA1 in a population-based series of American women.. JAMA.

[PDF_00693] Peto J., Collins N., Barfoot R., Seal S., Warren W., Rahman N., Easton D. F., Evans C., Deacon J., Stratton M. R. (1999). Prevalence of BRCA1 and BRCA2 gene mutations in patients with early-onset breast cancer.. J Natl Cancer Inst.

[PDF_00697] Petrij-Bosch A., Peelen T., van Vliet M., van Eijk R., Olmer R., Drüsedau M., Hogervorst F. B., Hageman S., Arts P. J., Ligtenberg M. J. (1997). BRCA1 genomic deletions are major founder mutations in Dutch breast cancer patients.. Nat Genet.

[PDF_00713] Ramus S. J., Kote-Jarai Z., Friedman L. S., van der Looij M., Gayther S. A., Csokay B., Ponder B. A., Olah E. (1997). Analysis of BRCA1 and BRCA2 mutations in Hungarian families with breast or breast-ovarian cancer.. Am J Hum Genet.

[PDF_00717] Roa B. B., Boyd A. A., Volcik K., Richards C. S. (1996). Ashkenazi Jewish population frequencies for common mutations in BRCA1 and BRCA2.. Nat Genet.

[PDF_00722] Struewing J. P., Abeliovich D., Peretz T., Avishai N., Kaback M. M., Collins F. S., Brody L. C. (1995). The carrier frequency of the BRCA1 185delAG mutation is approximately 1 percent in Ashkenazi Jewish individuals.. Nat Genet.

[PDF_00726] Struewing J. P., Hartge P., Wacholder S., Baker S. M., Berlin M., McAdams M., Timmerman M. M., Brody L. C., Tucker M. A. (1997). The risk of cancer associated with specific mutations of BRCA1 and BRCA2 among Ashkenazi Jews.. N Engl J Med.

[PDF_00730] Szabo C. I., King M. C. (1997). Population genetics of BRCA1 and BRCA2.. Am J Hum Genet.

[PDF_00631] Taylor-Papadimitriou J., Fentiman I. S. (1993). Breast cancer.. Cancer Surv.

[PDF_00732] Thorlacius S., Sigurdsson S., Bjarnadottir H., Olafsdottir G., Jonasson J. G., Tryggvadottir L., Tulinius H., Eyfjörd J. E. (1997). Study of a single BRCA2 mutation with high carrier frequency in a small population.. Am J Hum Genet.

[PDF_00742] Tonin P. N., Mes-Masson A. M., Futreal P. A., Morgan K., Mahon M., Foulkes W. D., Cole D. E., Provencher D., Ghadirian P., Narod S. A. (1998). Founder BRCA1 and BRCA2 mutations in French Canadian breast and ovarian cancer families.. Am J Hum Genet.

[PDF_00736] Tonin P., Weber B., Offit K., Couch F., Rebbeck T. R., Neuhausen S., Godwin A. K., Daly M., Wagner-Costalos J., Berman D. (1996). Frequency of recurrent BRCA1 and BRCA2 mutations in Ashkenazi Jewish breast cancer families.. Nat Med.

[PDF_00746] Xu C. F., Chambers J. A., Nicolai H., Brown M. A., Hujeirat Y., Mohammed S., Hodgson S., Kelsell D. P., Spurr N. K., Bishop D. T. (1997). Mutations and alternative splicing of the BRCA1 gene in UK breast/ovarian cancer families.. Genes Chromosomes Cancer.

